# Thoracic myopericytoma in an older adult, rare but possible: A case report

**DOI:** 10.1111/1759-7714.14535

**Published:** 2022-07-27

**Authors:** Maria Concetta Nigro, Maria Giulia Pirini, Elena Garelli, Marina Marchi, Alessandra Musto, Maria Abbondanza Pantaleo, Piergiorgio Solli, Andrea Ardizzoni, Margherita Nannini

**Affiliations:** ^1^ Department of Experimental, Diagnostic, and Specialty Medicine S.Orsola‐Malpighi University Hospital, University of Bologna Bologna Italy; ^2^ Pathology Unit S.Orsola‐Malpighi University Hospital Bologna Italy; ^3^ Division of Thoracic Surgery IRCCS Azienda Ospedaliera Universitaria di Bologna Bologna Italy; ^4^ Department of Radiology Bellaria/Bentivoglio Hospital Bologna Italy; ^5^ Nuclear Medicine Department Medicina Nucleare Metropolitana, Maggiore Hospital Bologna Italy; ^6^ Medical Oncology IRCCS Azienda Ospedaliero‐Universitaria di Bologna Bologna Italy

**Keywords:** myopericytoma, perivascular tumor, sub‐pleural lesion, thoracic myopericytoma

## Abstract

Myopericytoma is a rare tumor generally arising from skin and soft tissues of extremities, trunk, head, and neck regions, rarely from visceral sites. An intrathoracic visceral localization may carry a broad differential diagnosis including primary lung, pleura and chest wall lesions, or metastatic lesions. To date, any radiological features have been recognized and diagnosis of myopericytoma with intrathoracic localization remains still challenging. Here, we describe the case of a subpleural lesion incidentally diagnosed in an older adult affected by gastric cancer. Radiological features did not allow a differential diagnosis between a benign lesion, a primary tumor, or a metastasis. After resection, the histological examination showed histopathological features congruent with the diagnosis of myopericytoma. This unusual presentation reflects the need to share clinical, radiological, and histopathological data about this uncommon but frequently misdiagnosed disease.

## Graphical abstract

Myopericytoma rarely presents an intrathoracic localization. A well‐structured iconographic material is always an opportunity for all clinicians to assist in differential diagnosis with more frequent neoplastic lesions of lung, pleura, and chest wall, or other rare entities such as solitary fibrous tumor.

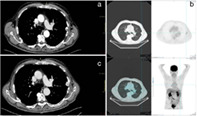

## INTRODUCTION

Myopericytoma is a rare tumor generally characterized by cells of different shapes arising from perivascular myoid cells, called myopericytes.^1^ It represents a quite recently delineated entity previously classified as a variant of hemangiopericytoma and currently classified by the World Health Organization (WHO) as soft tissue tumor, belonging to the group of peripheral blood cell/vascular cell tumors.^2^

The etiology is currently unknown, even if a co‐relation with trauma or viral infections, particularly Epstein–Barr virus (EBV) in patients with acquired immunodeficiency syndrome, has been suggested.^3^, ^4^

It is generally a tumor of childhood age, whereas it is uncommon in adults. It typically arises from distal extremities, occurring as single or multiple cutaneous or subcutaneous nodes, but it may also originate from other districts, such as the trunk, head, and neck, and occasionally from the visceral organs.^5^, ^6^, ^7^, ^8^, ^9^, ^10^, ^11^, ^12^, ^13^, ^14^, ^15^, ^16^

Histologically, myopericytomas are characterized by the presence of several blood vessels and a series of ovoid, plump, spiny, and/or round perivascular myoid cells, with eosinophilic cytoplasm.^1^, ^17^ Generally, a positive staining for h‐caldesmon, smooth muscle actin, and myosin heavy chain are frequently described, whereas immunostaining for desmin is often negative.^1^, ^17^

Most myopericytomas present as benign in nature with an indolent clinical course, even if a malignant behavior has been described in a few cases, usually occurring in deeper locations and sharing alarming morphological and immunophenotypic features such as the presence of an infiltrative growth, high mitotic activity, atypical cells, and necrosis.^10^, ^17^, ^18^

Here, we report a rare presentation of thoracic myopericytoma, diagnosed in an older male affected by gastric cancer.

### CASE REPORT

**FIGURE 1:**
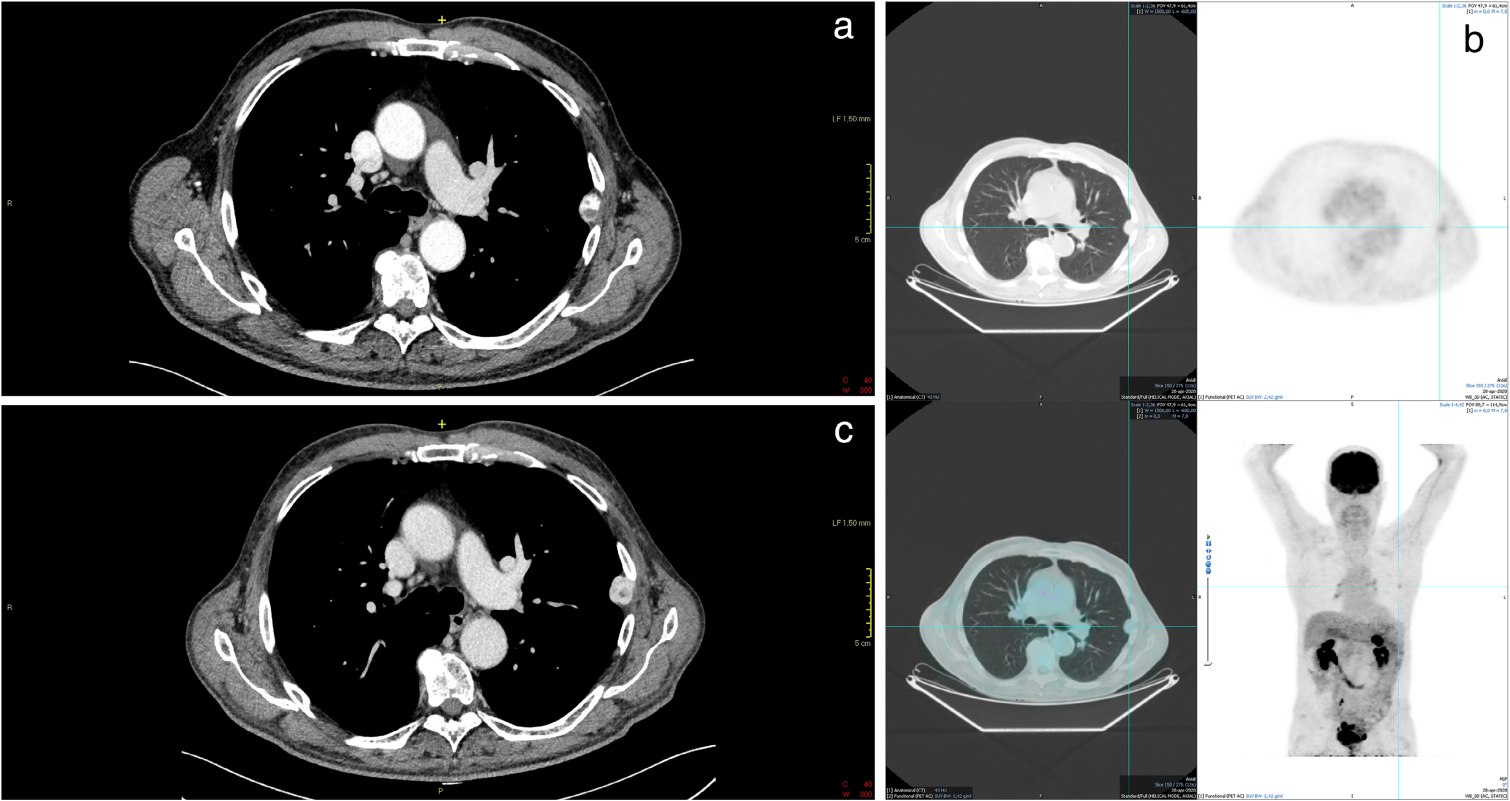
(a) Axial CT scan showing subpleural lesion of ~19 mm in size with regular margins and high contrast enhancement, arising from the thorax wall. (b) 18(F)‐fluorodeoxyglucose (FDG) positron emission tomography (PET) demonstrating a mild FDG uptake (SUVmax 2.4) of the thoracic lesion. (c) One‐year‐after axial CT scan showing the known subpleural lesion increased in size (24 × 22 mm), highly vascularized without signs of infiltration

In a 77‐year‐old male, an axial computed tomographic (CT) scan was performed during the radiological staging for a gastric cancer and showed a subpleural lesion of ~ 19 mm in size, with regular margins and high contrast enhancement, arising from the thorax wall (Figure 1(a)). At the subsequent 18(F)‐fluorodeoxyglucose (FDG) positron emission tomography (PET), the lesion presented a mild FDG uptake with uncertain significance (SUVmax 2.4) (Figure 1(b)).

One year later, the subpleural lesion has increased in size (24 × 22 mm), always highly vascularized, but without signs of infiltration (Figure 1(c)).

After multidisciplinary discussion, given the fact that primary or metastatic cancer could not be excluded, the patient underwent elective surgery. Complete resection of the lesion was performed by video‐assisted thoracoscopy. The postoperative course was uneventful and the patient was discharged 2 days after the intervention.

**FIGURE 2:**
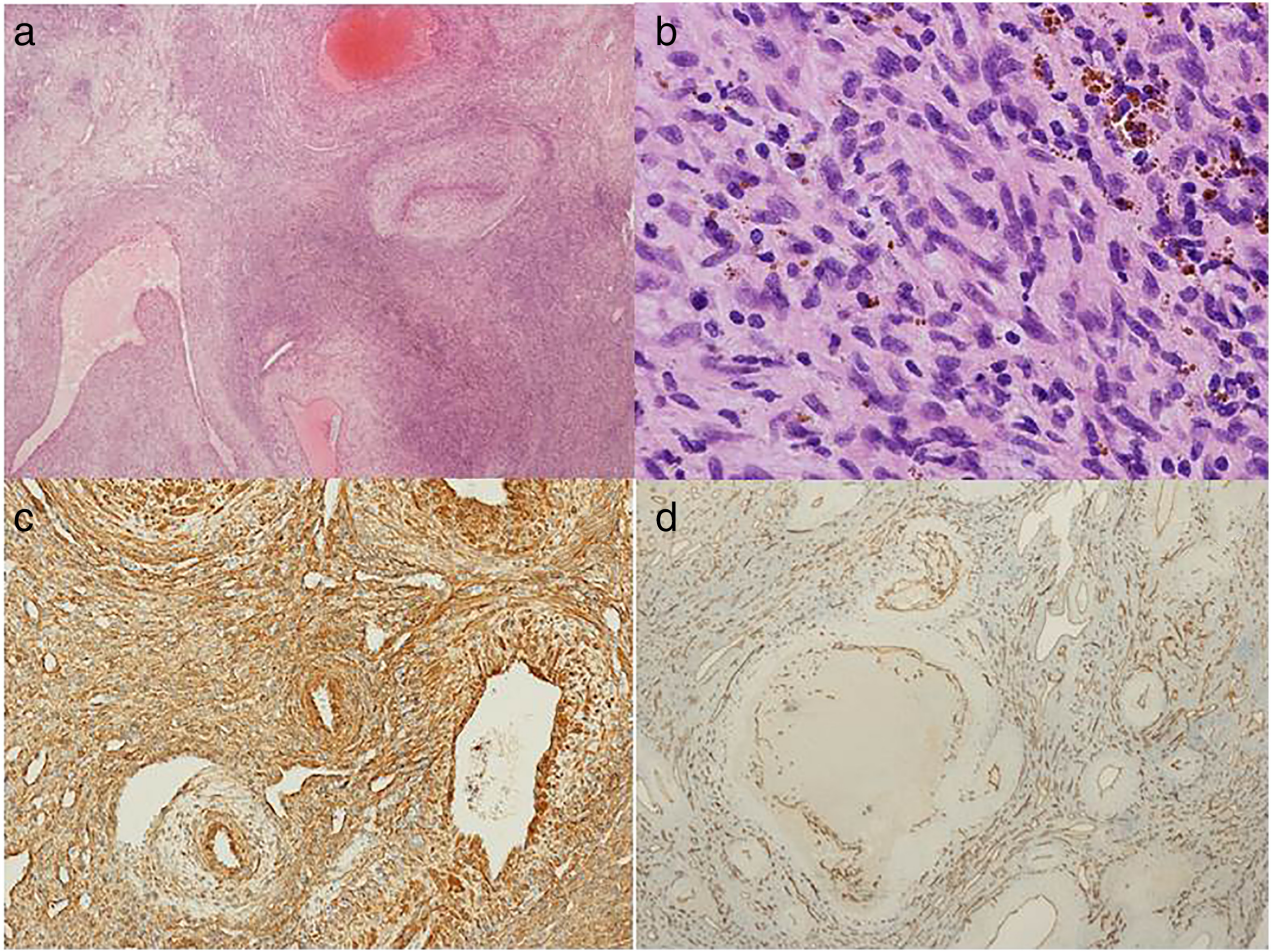
Histological findings. (a) The tumor was composed of blanching thick‐walled blood vessels with cellular stroma (Hematoxylin and Eosin stain (H&E) 10×). (b) The intermixed cellular stroma was composed of small spindled and ovoid cells with limited amounts of palely eosinophilic cytoplasm without atypia or pleomorphism. Deposits of hemosiderin were also present (H&E 40×). (c) The tumor cells were extensively positive for h‐caldesmon. (d) Staining for CD31 revealed a diffuse proliferation of variably sized blood vessels

Pathologic examination revealed a tumor consisting of, for the most part, small spindled or ovoid neoplastic cells with limited amounts of palely eosinophilic cytoplasm arranged around numerous delicate thin‐walled vascular channels, whereas, focally, there were larger myoid nodules with a whorled architecture and a hyaline stroma (Figure 2(a)). The larger nodules bulged into vascular lumina beneath an intact layer of endothelium. No significant atypia or pleomorphism was found (Figure 2(b)). Tumor cells were diffusely positive for h‐caldesmon, whereas desmin and STAT6 were negative (Figure 2(c)). Staining for CD31 highlights the delicate small vessels (Figure 2(d)). All these features fitted very well with a diagnosis of myopericytoma.

According to the uncertain malignant potential of the lesion because of the lack of significant atypia or pleomorphism, the patient continued with the periodic surveillance already ongoing for the gastric cancer.

## DISCUSSION

Myopericytoma represents a rare neoplastic entity with hemangiopericytoma‐like vascular pattern, mainly occurring in childhood to mid‐adult years, with a predilection for skin and soft tissues of the distal extremities. Less commonly, it can arise at other sites, including proximal extremities, head, neck, and trunk.^1^ Myopericytoma with an intrathoracic localization have been rarely reported. Clinic‐pathological features are summarized in Table 1.^6^, ^7^, ^8^, ^10^, ^11^, ^12^

**TABLE 1:** Summary of studies on primary intrathoracic myopericytomas

Reference	Localization	No. of patients	Age of patients (y)	Metastases at the diagnosis	Treatment approach	Recurrence after treatment	Outcome
Cao et al.^6^	Lower lobe of right lung	1	52	No	Lobectomy	No	Alive at 3 y
Edgecombe et al.^7^	Upper lobe of right lung	1	58	No	Wedge resection	No	Alive at 3 y
Song et al.^8^	Right and left lungs	1	26	No	Multiple pulmonary wedge resection	No	Alive at 3 y
Hodges et al.^10^	Pleura	1	57	No	Robotically assisted thoracic resection	NR	NR
Mun et al.^11^	Upper and lower lobe of left lung	1	63	No	VATS left lower lobectomy and upper wedge resection	No	Alive at 34 mo
Lombardi et al.^12^	Case 1. Lower lobe of left lung (subpleural site)	2	68	No	Partial resection	NR	NR
	Case 2. Right lobar bronchus (endobronchial site)		63	No	Right thoracotomy and lower lobectomy	NR	NR

Abbreviations: NR, not reported; No, number (of patients); Y, years; Mo, months; VATS, video‐assisted thoracoscopic surgery.

Limited data are available about radiological presentation of this type of neoplasm. In the ultrasound examination, the lesions appear homogeneously hypoechoic with well‐defined margins and clear blood flow.^19^ Although at conventional CT scans the lesions may have a wide spectrum of presentation (they can be hypodense or isodense, homogeneous or not, occasionally calcified, and with clear boundaries), at contrast‐enhanced CT evaluation myopericytoma appears as a homogeneous mass with complete uptake or just with peripheral enhancement that is typical of larger lesions with central necrosis.^6^, ^8^, ^15^, ^19^ At contrast‐enhanced MRI, myopericytoma presents intense enhancement. At conventional MRI, lesions generally appear hypointense in T1W1 scans, whereas in T2W1 scans they show a heterogeneous hyperintensity.^20^ This type of tumor presents a low‐moderate uptake of FDG at PET examination.^20^

Even if the high vascularization pattern, together with the low FDG uptake, may be pathognomonic findings, the radiological diagnosis of myopericytoma with intrathoracic localization remains still challenging. In fact, the viscelar localization is very unusual, especially in older adults, as in this clinical case, thus entering into differential diagnosis with the more frequent epithelial neoplastic lesions of the lung, pleura, and chest wall or with other rare entities such as solitary fibrous tumor, glomal tumors, and perivascular and mesenchymal tumors.

Therefore, an adequate histological and immunohistochemical characterization is mandatory for a definitive diagnosis and for identifying those rare cases with more aggressive features.

In summary, we report a rare case of intrathoracic myopericytoma made further unusual by the age of onset. Because radiological features appear to be not pathognomonic, it is important to spread awareness about this rare tumor among radiologists and clinicians, especially in the case of unusual presentations or clinical behavior.

## AUTHOR CONTRIBUTION

M.C.N. and M.N. collected, analyzed data, and wrote the case report. M.G.P. contributed to data curation (histological images). M.M. contributed to data curation (radiological images). A.M. contributed to data curation (radiological images). E.G., M.A.P., P.S., and A.A. reviewed and edited the manuscript. All authors read and approved the final manuscript.

## CONFLICT OF INTEREST

The authors declare that they have no conflicts of interests.

## Data Availability

All datasets generated for this study are included in the article.
